# Hot Topics and Frontiers of Resting-State fMRI in Parkinson's Disease: Research Trends and Paradigm Shifts From a Bibliometric Perspective

**DOI:** 10.1155/padi/6870215

**Published:** 2025-08-07

**Authors:** Yingni Jin, Jiayi Fu, Xiaojun Guan, Tao Guo, Xiaojun Xu

**Affiliations:** ^1^Department of Radiology, The Second Affiliated Hospital, Zhejiang University School of Medicine, Hangzhou 310009, China; ^2^Joint Laboratory of Clinical Radiology, The Second Affiliated Hospital, Zhejiang University School of Medicine, Hangzhou, China

**Keywords:** bibliometrics, CiteSpace, Parkinson's disease, resting-state fMRI, VOSviewer

## Abstract

**Background:** Parkinson's disease (PD), a progressive neurodegenerative disorder marked by motor and nonmotor symptoms, with resting-state fMRI (rsfMRI) proving pivotal in identifying neural circuit abnormalities and functional connectivity patterns, paving the way for a more personalized, precision medicine approach to PD diagnosis and treatment.

**Methods:** Given its significance, this study conducted a bibliometric analysis to systematically map the intellectual landscape of rsfMRI applications in PD research. Relevant publications were sourced from the Web of Science Core Collection database from January 1, 2009, to July 18, 2024, and restricted to English-language articles and review articles. Utilizing VOSviewer and CiteSpace software, the analysis covered publication distributions across countries, institutions, and authors, along with co-citation patterns among co-authors and journals, keyword co-occurrence, and burst detection.

**Results:** A total of 658 publications from January 1, 2009, to July 18, 2024, were identified, showing a gradual increase in annual publication and citation volume in earlier years. Notably, a decline emerged in 2023, highlighting the need for research paradigm shift to drive further progress. Among 45 countries and 865 institutions, China, led in publication and citation counts at both the institutional and author levels, with neuroscience-related journals playing a key role in advancing this field. Keyword analysis identified emerging research frontiers, including disease heterogeneity, early detection, symptom-specific mechanism exploration, and treatment evaluation.

**Conclusions:** Results from this bibliometric analysis systematically elucidates the historical development, research progress over the years, and current research hotspots in rsfMRI studies of PD, thereby offering valuable guidance for future research endeavors in this field.

## 1. Introduction

Parkinson's disease (PD) is a progressive multisystemic neurodegenerative disorder, characterized by α-synuclein aggregation and dopaminergic neurons loss in the substantia nigra pars compacta (SNpc) [1, 2]. These neuropathological changes lead to motor symptoms like bradykinesia, tremor, rigidity, and gait disturbances, along with nonmotor symptoms such as cognitive and psychiatric disturbances, which considerably impact individuals' quality of life and impose substantial socioeconomic and healthcare burdens [3, 4]. The specific biological basis and neural mechanisms of PD remain unclear, despite the fact that substantial efforts have been made to identify its pathogenic factors, clinical manifestations, and therapeutic strategies.

In recent years, resting-state functional magnetic resonance imaging (rsfMRI) has advanced our understanding of the neural pathophysiology of PD, improving diagnostic precision and enabling more personalized therapeutic strategies. Initially, rsfMRI studies focused on basic brain indicators, such as amplitude of low-frequency fluctuation (ALFF) for local activity intensity, regional homogeneity (ReHo) for local neural synchronization, and functional connectivity (FC) for inter-regional interactions and information exchange. These approaches identified spontaneous brain activity patterns, including the characterization of intrinsic functional networks (e.g., default mode network) and localized dysfunction and hyperconnectivity in neuropsychiatric disorders [5, 6]. Subsequently, graph theoretical analysis emerged, conceptualizing the brain as a complex network. This method allowed researchers to quantify both global properties (e.g., small-worldness) and nodal characteristics (e.g., hub distribution). By analyzing the attributes of brain network, research showed that PD patients exhibit significant network disruption, marked by decreased clustering coefficients and local efficiency despite the preservation of small-world topology [7], which likely underlies their cognitive and motor impairments. Further methodological advancements introduced dynamic FC (dFC) [8], which captures time-varying interactions among different brain regions and their transition. Kim et al. [8], employing dynamic FC analysis, revealed PD patients exhibited significantly reduced functional segregation in brain networks, accompanied by a marked increase in the temporal variability of network efficiency.

Despite broad recognition of rsfMRI's potential in PD, many challenges and uncertainties remain, including clinical heterogeneity at the individual level, methodological complexity and challenge of molecular biological interpretability in rsfMRI and the lack of reliable neuroimaging biomarkers for predicting disease progression and treatment outcomes [9, 10]. Currently, it is both imperative and suitable to systematically summarize and analyze the research status, as it would deepen our understanding of the main progress, pinpoint research hotspots and frontiers, and evaluate the impact of prior work, providing valuable guidance for future research endeavors.

Bibliometrics, a widely recognized and rigorous methodology for literature analysis, employs quantitative approaches to analyze and track research development in specific fields [11]. Tools like VOSviewer and CiteSpace enable the visualization of complex data through the construction of network maps, revealing structural patterns, collaborative networks, and research trends. Consequently, the primary purpose of this work was to perform a comprehensive review utilizing bibliometric analysis to clarify the historical evolution, current research state, and prospective future directions of rsfMRI studies related to PD.

## 2. Methods

### 2.1. Data Collection

**Figure 1:**
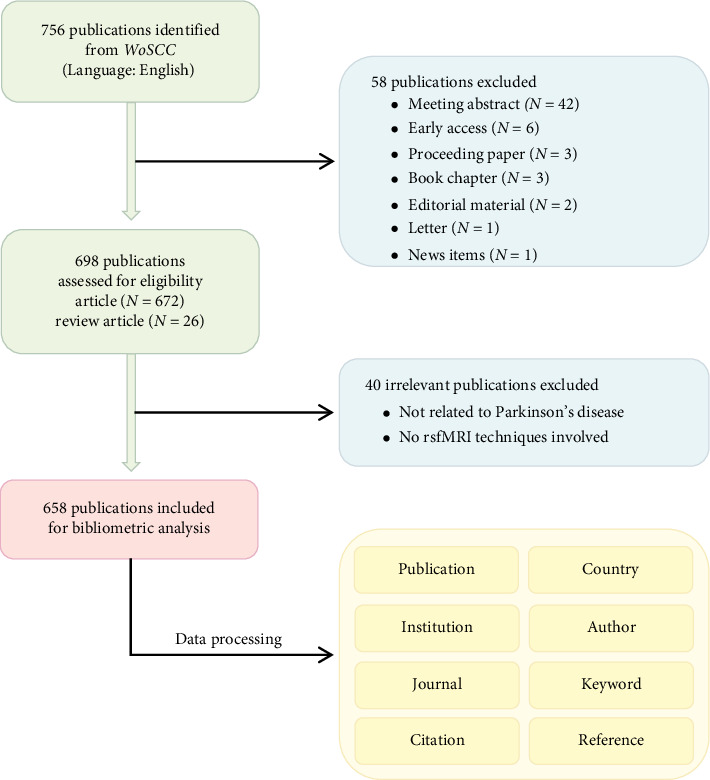
Flowchart for searching and selecting literature.

The Web of Science Core Collection (WoSCC), known for its extensive repository of high-quality scientific literature, served as the source database for data retrieval. Relevant studies were identified on July 18, 2024. Although WoSCC database covers publications from 1985 to 2024 by default, the earliest relevant records in our analysis date to 2009. To maintain temporal relevance, we restricted the literature selection to articles published between January 1, 2009, and July 18, 2024. The search strategy was delineated as follows: TS = (“resting-state” or “rest” or “resting”) AND TS = (“functional magnetic resonance imaging” or “functional MRI” or “fMRI”) AND TI = (“Parkinson” or “Parkinson's disease” or “PD”). The search was limited to English-language articles and review articles. Other document types, such as meeting abstract, early access material, proceedings paper, book chapter, editorial material, letter, and news item, were excluded. Following duplicate removal, titles, abstracts, keywords, and full texts (when necessary) were manually screened by two experienced researchers to identify studies that met our inclusion criteria: (1) focused on PD; (2) rsfMRI methodology. For any literature with discrepancies, the researchers would reach a consensus through discussion or third-party opinions if necessary. Finally, the complete records and cited references for each eligible publication, including title, authors, source, times cited counts, and keywords were exported as plain text files. The whole search process is present in Figure 1.

### 2.2. Bibliometric Analysis

This study utilized VOSviewer (version 1.6.20) and CiteSpace (version 6.3.R1) for bibliometric visualization and quantitative analysis of published literature. Key analyses included annual publication trends, collaborative networks (e.g., countries, institutions, and authors), along with sources and authors co-citation patterns keyword clustering analysis, and burst detection. Co-citation analysis maps relationships between cited works by analyzing how often two items (e.g., authors, references, or journals) are cited together [12]. After importing data from the WoSCC database and selecting the co-citation analysis option, VOSviewer generates a network in which nodes correspond to these items and links indicate their co-citation frequency. This analytical approach enables researchers to uncover the connections and mutual influences among academic publications or authors. Keyword clustering analysis in VOSviewer identifies distinct thematic groups that capture key research trends [13]. To enhance the clarity of these themes, we merged synonymous terms and subsequently performed the initial visualization based on a minimum occurrence frequency of 10. The layout settings were adjusted to enhance graphic readability, with nodes representing keywords and colors indicating their respective clusters. Concurrently, burst detection in CiteSpace identifies terms with sudden surges in frequency to uncover shifts in research focuses and emerging trends over time within related fields [14]. In visualization maps, nodes represent items (e.g., countries, institutions, authors, and keywords), with their size indicating occurrence frequency and color corresponding to cluster affiliation. Connecting lines signify links between nodes, with line thickness reflecting connecting strength. Moreover, total link strength (TLS), automatically generated by VOSviewer, quantifies the cumulative connection strength between nodes within the constructed networks [15, 16]. In addition, Microsoft Office Excel (2021) facilitated qualification, while Origin (2021) generated the chord diagram.

## 3. Results

### 3.1. Annual Publications and Citations

**Figure 2:**
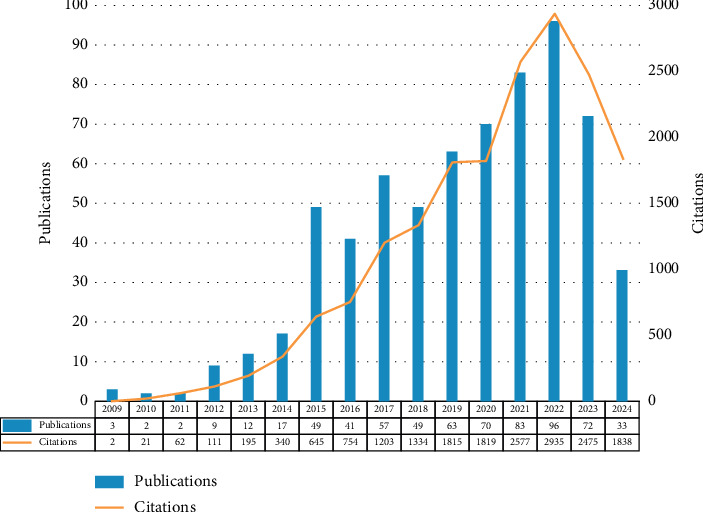
Annual publications and citations trend in rsfMRI research related to Parkinson's disease from January 1, 2009, to July 18, 2024.

A total of 658 publications selected in this study were written by 3185 authors affiliated with 865 organizations across 45 countries, published in 141 journals, and cited 16,023 citations from 178 journals. By analyzing the temporal trends of annual publications and citations in the data and chart (Figure 2), we empirically determine the three distinct developmental stages: the initial stage (2009–2013), rapid developmental stage (2014–2022), and the late stage (2023–2024). Between 2009 and 2013, there remained less than 20 publications and 400 citations annually. However, since 2014, the field has undergone rapid development and peaked in 2022 with 96 articles and 2935 citations. This trend suggested the growing interest in applying rsfMRI to PD research. In the latter phase, the number of papers published in 2023 dropped to 72 with 2475 citations, even less than in 2021, suggesting possible saturation with decreased interest. Although the data for 2024 remains incomplete, with the search ending on July 18, 2024, current indicators point to a continued downward trend compared with previous years, signaling the potential need for methodological innovation or research paradigm shifts in the field.

### 3.2. Analysis of Country/Region

**Figure 3:**
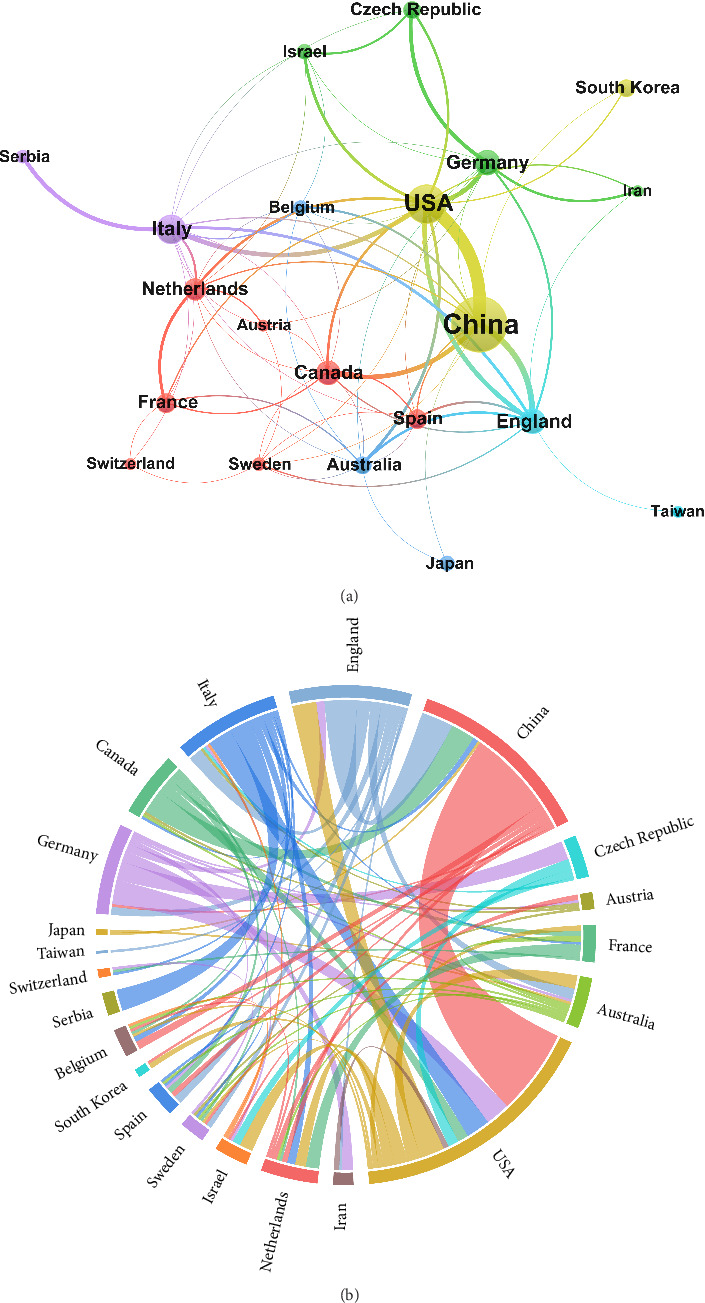
Country/region collaboration map and chord diagram in rsfMRI research associated to Parkinson's disease. (a) Country/region collaboration map. Each node represents a country or region with its size indicating the number of publications. Lines between nodes signify collaborative efforts on at least one publication, with the thickness corresponding to the degree of collaboration between them. (b) Chord diagram. Each node represents a country/region. The nodes are arranged radially along a circle, with their width proportional to the number of publications. Nodes are interconnected by weighted arcs, indicating that they share at least one common publication, with thicker arcs reflecting a higher number of shared publications.

The published literature originated from 45 countries or regions, with the top 15 countries/regions are listed in Table 1. China had the largest number of publications with 329 articles, accounting for nearly half of total publications, followed by the United States (*n* = 131) and Italy (*n* = 60). In terms of citation counts, China consistently led with 6025 citations, followed by the United States with 3925 and Italy with 2318. The United States exhibited the strongest TLS of 104, followed by China (71) and the United Kingdom (47).  Figure 3(a) illustrates collaboration networks among 21 countries or regions, each having at least five relevant publications. The chord diagram (Figure 3(b)) further visualized the mutual cooperation among these countries, such as China with Canada and England, and the United States with Germany. What stands out in it is the thickest arc linking China and the United States, reflecting their tight cooperative interactions in this domain.

### 3.3. Analysis of Institution

**Figure 4:**
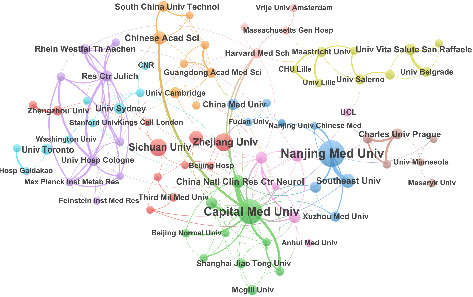
Institution collaboration map. Each node represents an institution with node size indicating the number of publications. Lines between nodes signify collaborative efforts on at least one publication, with the thickness corresponding to the degree of collaboration between them.

The top seven institutions in Table 2 are geographically located in China, with the remaining three distributed across Germany, England, and Italy, respectively. Nanjing Medical University ranked first with 60 publications, followed by Capital Medical University (*n* = 52) and Chinese Academy of Sciences (*n* = 28). Capital Medical University achieved the highest citation count with 1625 citations and the strongest TLS of 133, indicating its pivotal role in institutional collaboration.  Figure 4 illustrates institutional collaborative network, which included 81 out of the 865 institutions, applying a threshold of at least five publications. As the leading institution, Nanjing Medical University demonstrated close cooperative relationships with other institutions, including Southeast University, Nanjing University of Chinese Medicine, and Peking University.

### 3.4. Analysis of Authors and Co-Cited Authors

**Figure 5:**
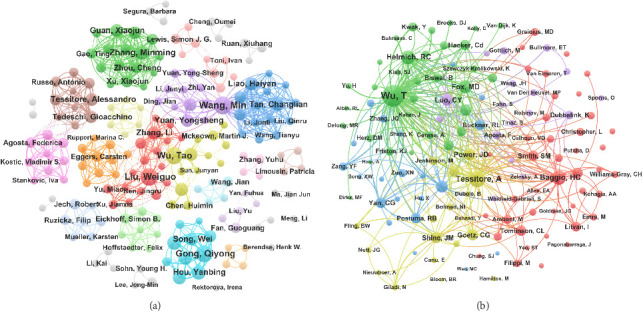
Visualization map of authors and co-cited authors. (a) Collaborative network knowledge map of author. Each node represents an author with node size indicating the number of publications. Lines between nodes signify collaborative efforts on at least one publication, with the thickness corresponding to the degree of collaboration between them. (b) Collaborative network knowledge map of co-cited author. Each node represents an author with node size indicating times he/she has been cited. Lines between nodes signify the frequency with which their published articles have been co-cited by a third article, with thicker lines denoting a higher frequency of co-citation.

The author's analysis presented information regarding the core research strengths and representative scholars within the field. The top 15 most authoritative authors are shown in Table 3, among whom Wang Min and Gong Qiyong took the lead with 25 publications each, followed closely by Liu Weiguo (*n* = 24) and Wu Tao (*n* = 23). Wu Tao received the highest citation count with 1172 citations and Liu Weiguo had the strongest TLS of 236. The co-authorship network of those with at least 5 publications is present in Figure 5. Numerous research teams were identified, characterized by robust internal collaboration within teams but relatively limited inter-team cooperation.

Co-citation analysis is generally regarded as a better method to evaluate a scholar's academic influence within their field. Of the 10,288 co-cited authors, 12 exceeded 150 co-citations (Table 4). Wu Tao was the most cited author of co-citations (492) with his work primarily investigating FC alterations and identifying potential biomarkers for early diagnosis and therapeutic monitoring in PD patients.

### 3.5. Analysis of Journal and Co-Cited Journal

**Figure 6:**
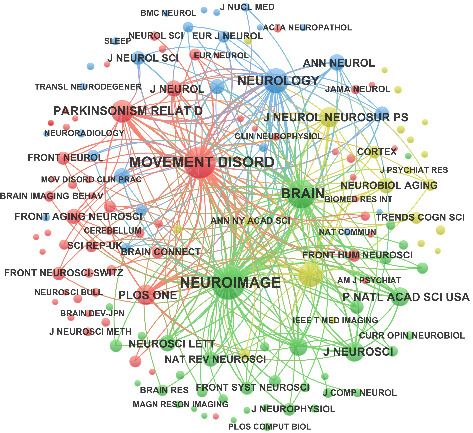
Visualization map of co-cited journals. Each node represents a journal with node size indicating times it has been cited. Lines between nodes signify the frequency with which their published articles have been co-cited by a third journal with thicker lines denoting a higher frequency of co-citation.

Table 5 illustrates the top 15 productive journals in rsfMRI research related to PD. Most journals specialize in neuroscience or its subfields with only a few exceptions being comprehensive journals. The leading 9 journals each contributed over 20 publications, with Frontiers in Aging Neuroscience at the forefront (*n* = 49), followed by Parkinsonism and Related Disorders (*n* = 38), Human Brain Mapping (*n* = 33), Movement Disorders (*n* = 33), and Frontiers in Neuroscience (*n* = 32). According to the 2024 Journal Citation Reports (JCR), eight of these journals in Table 5 are classified in Q1 and five in Q2, reflecting their high academic impact. As shown in Table 6, the three most-cited journals each accumulated over 2000 co-citations with Neuroimage reaching 3051. All of these were categorized in JCR Q1, indicating their prominence in this field.  Figure 6 presents the co-citation network of journals with at least 30 co-citations.

### 3.6. Analysis of Keywords

**Figure 7:**
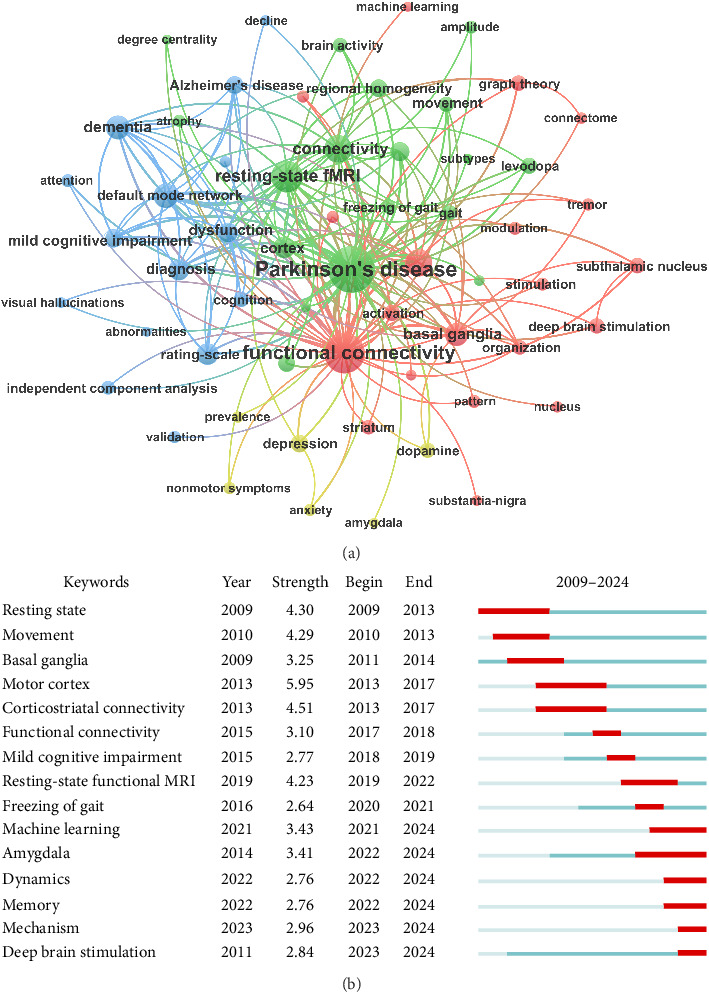
Keyword co-occurrence analysis. (a) Keyword co-occurrence visualization network. Each node represents a keyword with node size indicating the frequency of its appearance in the literature. Lines between nodes signify that the corresponding keywords co-occur within the same article with thickness representing the frequency of their co-occurrence. The thicker the line, the more commonly they appear together, showing their stronger correlation. (b) Top 15 strongest theme terms in the burst detection. “Keywords” in the first column denotes the identified burst terms, “year” in the second column specifies the initial year of their emergence, “strength” denotes the burst intensity of the terms, “begin” signifies the year of the term outbreak, and “end” marks the concluding year of the burst. The burst end year for the final few keywords was 2024 because the search period concluded in 2024. Light blue segments indicate nonburst periods, where a keyword is mentioned with low frequency or prominence, without reaching significant burst intensity. Red segments signify terms experiencing rapid growth during a specific time period, while dark blue lines suggest that the research field may be undergoing a recession or has reached a stable stage.

Following synonyms merging keyword co-occurrence network was constructed with those appeared more than 10 times and subsequently segmented it into 4 clusters (Figure 7(a)). Cluster 1 (red part, including 20 items) emphasized the neural circuit mechanism and neural regulation, highlighting keywords such as “functional connectivity,” “networks,” “basal ganglia,” “deep brain stimulation,” “subthalamic nucleus,” and “graph theory.” Cluster 2 (green section with 18 items) was primarily related to disease heterogeneity and motor-related terms, underscoring terminologies like “cortex,” “progression,” “movements,” “freezing of gait,” “levodopa,” and “subtypes.” Cluster 3 (blue, with 15 items) mainly consisted of cognition-related terminology with keywords including “dementia,” “default mode network,” “mild cognitive impairment,” “dysfunction,” “rating-scale,” and “independent component analysis.” Cluster 4 (yellow, comprising 6 items) focused on emotional symptoms, covering terms such as “depression,” “anxiety,” “amygdala,” and “dopamine,”

Burst thematic terms, which play a crucial role in identifying emerging trends, often act as key indicators of frontiers research hotspots. We utilized CiteSpace to identify the top 15 keywords with the strongest burstiness (Figure 7(b)), where the term “motor cortex” exhibited the strongest burst strength of 5.95. Early neuroimaging research predominantly examined the functional role of discrete brain regions in PD, particularly structures such as the basal ganglia, motor cortex, and limbic system (e.g., the amygdala) in fundamental motor control and memory processes. More recent work has instead conceptualized the brain as large-scale integrative networks, highlighting the critical role of connectivity and dynamic interactions between regions. Advances in machine learning and multimodal neuroimaging have enabled more sophisticated analyses of brain networks, supporting the identification of biomarkers and the development of personalized therapeutic strategies. These advancements bridged the gap between basic neuroscience and clinical applications, offering new avenues for future research.

## 4. Discussion

### 4.1. Current Research Status

This bibliometric analysis of PD research with the application of rsfMRI from January 1, 2009, to July 18, 2024, yielded a substantial amount of information. The publication and citation volume evolved through the three divided stages: an initial phase of slow growth, followed by a period of rapid expansion, and ultimately transitioning into a phase marked by a potential declining trend, underscoring the dynamic trajectory of research progress. The initial growth phases reflected the establishment and development of the field, while the recent decline may indicate that rsfMRI research in PD has reached a bottleneck that failed to find groundbreaking discoveries, possibly attributed to the inherent heterogeneity of PD and limitations of current research paradigm. Moreover, as the field has matured, research may be shifting towards more focused and specialized areas, leading to the stabilization or even reduction in overall publication output.

The collaboration networks among countries, institutions, and authors reflect the geographical distribution of research output. China and the United States dominated the field, as evidenced by their substantial volumes of publications and citations. This prominence is likely due to greater access to data and resources, together with strong institutional and academic support for scientific advancements. Furthermore, the observed pattern of intensive intra-team collaboration but limited inter-team cooperation may impede innovation by restricting knowledge and resource exchange. Strengthening inter-team communication, encouraging cross-disciplinary projects, and establishing dedicated platforms for knowledge and resource sharing would mitigate these limitations and enhance cross-team collaboration and promote further progress in this field.

### 4.2. Research Hotspots and Frontiers

The evolution of keyword reflects the structural and dynamic progression within a knowledge domain. Based on keyword co-occurrence analysis and burst detection, we identified four research hotspots and emerging frontiers in rsfMRI studies related to PD, as described below.

#### 4.2.1. Disease Heterogeneity in PD

PD exhibits significant clinicopathologic heterogeneity, with substantial variations observed in clinical manifestations, disease progression, therapeutic response, and neuropathological changes, suggesting the presence of distinct disease subtypes [17].

Conventional classification paradigms in PD primarily distinguish subtypes based on either age of onset [18, 19], typically demarcated at 40 or 50 years, or motor phenotypes [20], which categorize PD into tremor-dominant (TD) and postural instability and gait difficulty-dominant (PIGD) subtypes. Emerging neuroimaging evidence has highlighted the significant roles of cerebellum and thalamus in distinguishing PD motor subtypes [21, 22], with longitudinal studies revealing dynamic subtype transitions [23]. Basaia et al. identified a transition from TD to PIGD linked to alterations in the cerebro-cerebellar motor network [24]. In addition, related studies have proposed classifications dominated by nonmotor symptoms, including cognitive impairment [25, 26], affective disorders [27, 28], and rapid eye movement sleep behavior disturbances [29]. Nevertheless, these classifications depend on specific clinical symptoms and fail to account for integrating multidimensional symptoms and neurobiological correlates in PD.

Contemporary hypothesis-free, data-driven methodologies have increasingly been applied to define subgroups. Recent unsupervised clustering analysis [30] categorized PD into mild motor predominant, intermediate, and diffuse malignant subtypes based on comprehensive clinical measurements, revealing differential neurodegeneration patterns and prognostic differences [31, 32]. Similarly, Guo et al. [33] identified three PD subtypes based on the clinically relevant connectivity patterns, in which the severe depression-dominant subtype exhibited widespread disruptions both in brain function and structure. Recent advances in biological classification frameworks for PD have shifted subtyping strategies toward a more biologically grounded approach [34, 35], integrating aggregated α-synuclein and its associated neurodegeneration to better capture molecular and biological-level disease heterogeneity. However, the proposed biological frameworks are still in the clinical research stage, necessitating further validation and refinement of them prior to wider application in clinical research and potential translation into clinical practice. While data-driven approaches have been widely applied for PD subtyping, the impact of these identified subtypes on our understanding of the pathological mechanism and treatment of PD remains unclear with poor clinical applicability and reproducibility [36]. Future efforts in PD subtyping should prioritize the development of biomarker-driven frameworks, improving the precise subtype classification algorithms, and the validation of these classifications across diverse cohorts to ensure reproducibility [37]. Such advances will be pivotal in elucidating PD pathology and ultimately promoting therapeutic strategies.

#### 4.2.2. Early Diagnosis of PD

rsfMRI has emerged as a powerful tool for early detection of PD through identifying subtle alterations in brain FC that often precede clinical manifestations, enabling timely intervention and thereby mitigating disease progression [38–40].

Significant progress has been made in early detection for differentiating PD patients from healthy controls. A connectome-level study revealed disrupted basal ganglia connectivity in PD group, particularly with the sensorimotor, default mode, and visual networks, aligning closely with the connectome spreading-based model of brain pathology [41]. Utilizing rsfMRI data, Islam et al. [42] achieved 86.07% validated accuracy employing 3D convolutional neural networks for PD diagnosis. Xu et al. [43] identified low-dimensional graph-theoretical features of FC as potential neuroimaging biomarkers, with accuracy up to 96.4%. Multimodal integration approaches combining structural MRI with rsfMRI have shown promise, though with more modest classification accuracy of 75% [44].

Differentiation of PD from multiple system atrophy (MSA) in the early stages remains clinically challenging due to overlapping clinical manifestations and the absence of specific biomarkers. Wang et al. [45] validated the efficacy of fractional ALFF in the putamen to discriminate PD from MSA, consistent with the findings reported by Hou et al. [46]. Chen et al. [47] found disrupted basal ganglia-cortical connectivity in PD patients and significant cerebellum-cortical disconnection in those with MSA, and the validation study showed a regularized logistic regression classifier outperformed conventional machine learning approaches, achieving a discriminative accuracy of 92.31%.

The existing research on diagnosis has achieved valuable results, but discrepancies in reported diagnostic accuracy and specificity of early-stage PD persist, possibly due to several potential factors. The variation in data stemming from diverse ethnic populations, different age groups, and varying disease stages may introduce bias into the results and constrain the generalizability of the classification models [48]. Meanwhile, methodological variations in feature extraction, classifier selection, and whether to perform external validation or cross-data validation further contribute to outcome variability. Resolving these challenges could advance the advancement of dependable and clinically applicable tools for early and precise PD diagnosis [49].

#### 4.2.3. Neuropathological Mechanisms of PD

Advancements in rsfMRI have enabled precise characterization of large-scale network reorganization in PD, with growing evidence linking specific connectivity patterns, such as functional decoupling and hyperconnectivity, to its motor and nonmotor manifestations [50, 51].

Motor symptoms, such as tremor and freezing of gait (FOG), represent hallmark manifestations of PD. The “dimmer-switch” hypothesis [52] posits that tremor originates in the basal ganglia while the cerebello-thalamo-cortical circuit functions as an amplitude modulator. Shen et al. [53] validated this model by demonstrating cerebellar-sensorimotor hyperconnectivity, indicative of compensatory network reorganization. Similarly, studies on FOG implicated critical involvement of multiple neural structures, including the basal ganglia [54, 55], cerebellum [56, 57], and brainstem [58, 59], in control of human posture and gait performance. Furthermore, converging evidence has confirmed the impaired FC between the right fronto-parietal and executive-attention networks, associated with FOG severity [60].

Beyond conventional motor dysfunction, cognitive impairment represents a highly prevalent nonmotor feature of PD. Previous neuroimaging studies have consistently demonstrated reduced FC in sensory-motor networks, including the sensorimotor, visual, and auditory network, in PD patients with mild cognitive impairment (MCI) [61, 62]. Findings regarding cognitive-related networks, however, have yielded variations, and sometimes contradictory outcomes regarding connectivity changes [63]. For instance, Hou et al. [64] and Suo et al. [65] reported decreased nodal centrality within the DMN in early-stage PD-MCI patients, while Chen et al. [66] observed increased nodal centrality in the same network. These divergent findings confirmed the complexity of cognitive network dysfunction in PD which potentially reflected differential compensation patterns across disease stages and warranted further investigation. The neuropsychiatric comorbidities of PD, such as depression and anxiety, exhibit distinct neural circuit signatures. Emerging evidence reported PD with depression manifests dysfunctional prefrontal-limbic integration [67, 68], whereas anxiety symptoms correlate with amygdala hyperactivity and striatal-limbic network reorganization [69, 70].

Despite these advances, the complexity of functional networks and the heterogeneity of symptom manifestations in PD often involve multinetwork interactions or compensatory mechanisms [71]. Whether such alterations can precisely reflect specific symptom changes and the molecular biological mechanisms underlying them remains incompletely understood, which partially limit the utility of rsfMRI in elucidating symptom-specific pathology in PD. To uncover the biological basis of network disruptions and identify disease-specific molecular signatures, further studies should integrate FC changes to molecular features, such as gene expression data from the Allen Human Brain Atlas and neurotransmitter expression data from the neurotransmitter atlas. To ensure the robustness and generalizability of these findings, multicenter studies with sufficiently large sample sizes and rigorous stratification based on disease severity, duration, and specific symptomatology, combined with methodological refinements, are essential to more precisely delineate the relationship between functional network reorganization and specific symptoms in PD [72].

#### 4.2.4. Treatment Assessment in PD

The application of rsfMRI provides a valuable framework for evaluating the efficacy of therapeutic interventions, including pharmacological treatments and neuromodulation, enabling the optimization of therapeutic strategies [73, 74].

Current anti-PD pharmacotherapy relies mainly on dopamine replacement therapies as first-line treatment, complemented by nondopaminergic agents. Serving as the dopamine precursor, levodopa supplements dopamine deficiency, demonstrating efficacy in mitigating disease progression and alleviating PD-associated complications [75]. Dopamine replacement therapy dynamically modulates dysregulated brain networks, effectively normalizing aberrant connectivity patterns [76, 77]. Wu et al. [78] indicated dopamine normalizes hyperconnectivity in motor-related networks while strengthening connectivity in salience and frontal networks, which correlates with better motor performance, revealing distinct connectome patterns underlying dopaminergic therapy. Subsequent studies confirmed levodopa's capacity to restore pathological network topology, normalizing the SMN for motor symptom alleviation [79] and modulating DMN dysregulation to enhance cognitive performance [80]. However, long-term levodopa administration carries risk of symptom fluctuations and levodopa-induced dyskinesia (LID), with emerging evidence suggesting ventral pallidum-related network involvement in LID pathogenesis [81]. These findings underscore the necessity for continued investigation into LID neurocircuitry and precision therapeutic strategies.

Neuromodulation techniques such as DBS and repetitive transcranial magnetic stimulation (rTMS) stimulate specific brain regions to alleviate symptoms and improve quality of life [82, 83]. DBS modulates cortico-basal ganglia-thalamic circuits, with rsfMRI evidence showing enhanced thalamo-cortical connectivity that correlates with motor improvement [84, 85]. Longitudinal studies comparing high- and low-frequency stimulation of the subthalamic nucleus identified two distinct neural circuits, each associated with specific symptom profiles [86]. Similarly, rTMS improves motor symptoms like bradykinesia by modulating cerebellar-thalamic neural activity [87]. Despite these advances, current neuromodulation techniques face several unresolved challenges spanning mechanistic ambiguities and procedural optimization. Key problems include precise target selection, accurate intraoperative localization, postoperative parameter optimization, and adverse effect mitigation. rsfMRI show clinical potential by mapping individualized functional networks which are further analyzed using data-driven neural network models to facilitate stimulus target selection and stimulation parameter optimization, paving the way for the development of personalized and effective treatment strategies in PD [88].

## 5. Limitations

This study has several limitations. Initially, while relying solely on WoSCC may have omitted important literature from other databases, this platform remains a globally recognized authoritative source that captures major research trends in this field. To avoid duplication, our study focused exclusively on articles and review articles written in English, ensuring consistency in analysis and interpretation. Furthermore, this study only included publications up to the search date; possibly ignoring some most-recently published high-quality publications and resulting in incomplete research findings. Future studies are essential to continuously track the latest data and research trends to compensate for this limitation.

## 6. Conclusions

This bibliometric analysis presents global collaborative relationships among scholars, institutions, and countries in advancing rsfMRI applications for PD. While earlier studies demonstrated a general upward trend, the observed decline in 2023 suggests the need for methodological innovation and research paradigm shift to drive further progress. To effectively translate rsfMRI findings in PD into clinical applications, future research should focus on refining disease subtyping and diagnostic accuracy, clarifying disease mechanism of PD at both neuropathological and molecular biology level, and developing clinically relevant biomarkers for monitoring treatment efficacy. These efforts hold the potential to deepen our understanding of PD pathophysiology and facilitate the clinical translation of rsfMRI for clinical diagnosis, progression monitoring, and therapeutic evaluation.

## Figures and Tables

**Table 1:** Top 15 countries/regions in terms of publication outputs in the collaborative network.

Index	Country/region	Count	Citation	Total link strength
1	China	329	6025	71
2	United States	131	3925	104
3	Italy	60	2318	38
4	Germany	42	1500	35
5	England	39	1610	47
6	Canada	38	1348	28
7	Netherlands	28	1800	20
8	Spain	21	1138	13
9	France	21	646	14
10	Czech Republic	17	428	17
11	South Korea	17	325	3
12	Australia	16	541	22
13	Japan	11	138	2
14	Israel	10	180	14
15	Belgium	9	227	10

**Table 2:** Top 10 institutions in terms of publication outputs in the collaborative network.

Index	Institution	Count	Citation	Total link strength
1	Nanjing Medical University (China)	60	928	61
2	Capital Medical University (China)	52	1625	133
3	Chinese Academy of Sciences (China)	28	251	54
4	Zhejiang University (China)	27	415	26
5	Sichuan University (China)	26	733	48
6	Southeast University (China)	18	428	37
7	Central South University (China)	18	102	18
8	Helmholtz Association (Germany)	17	354	26
9	University of Cologne (England)	16	577	82
10	Università degli Studi della Campania Luigi Vanvitelli (Italy)	16	245	35

**Table 3:** Top 15 authors in terms of publication outputs in the co-authorship network map.

Index	Author	Count	Citation	Total link strength
1	Qiyong Gong	25	729	194
2	Min Wang	25	405	231
3	Weiguo Liu	24	404	236
4	Tao Wu	23	1172	148
5	Huifang Shang	22	627	158
6	Yongsheng Yuan	21	368	156
7	Kezhong Zhang	21	368	156
8	Minming Zhang	18	371	219
9	Jing Yang	16	604	112
10	Yanbing Hou	16	254	106
11	Piu Chan	15	1002	90
12	Tao Feng	15	279	103
13	Ruwei Ou	15	250	105
14	Alessandro Tessitore	14	1131	112
15	Wei Song	14	577	101

**Table 4:** Top 15 cited authors based on co-citations in the visualization map of co-cited authors.

Index	Author	Co-citation	Total link strength
1	Tao Wu	492	28,714
2	Alessandro Tessitore	278	16,353
3	Rick C. Helmich	206	12,994
4	Andrew J. Hughes	199	10,638
5	Hugo Cesar Baggio	186	11,773
6	Christopher G. Goetz	176	10,167
7	Michael D. Fox	174	10,485
8	Ronald B. Postuma	171	9954
9	Chunyan Luo	166	9288
10	Heiko Braak	163	10,488
11	Chaogan Yan	163	8778
12	Jonathan D. Power	162	10,245
13	James Mac Shine	149	8894
14	Claire L. Tomlinson	141	8172
15	Dag Aarsland	133	7493

**Table 5:** Top 15 productive journals related to the resting-state fMRI research in PD.

Index	Journal	Publication	IF (2024)	JCR division
1	Frontiers in Aging Neuroscience	49	4.1	Q2
2	Parkinsonism and Related Disorders	38	3.1	Q2
3	Human Brain Mapping	33	3.5	Q1
4	Movement Disorders	33	7.4	Q1
5	Frontiers in Neuroscience	32	3.2	Q2
6	Frontiers in Neurology	31	2.7	Q3
7	Neuroimage-Clinical	29	3.4	Q2
8	PLoS One	23	2.9	Q1
9	CNS neuroscience and therapeutics	21	4.8	Q1
10	Neuroscience Letters	19	2.5	Q3
11	Brain Imaging and Behavior	19	2.7	Q2
12	Brain	17	10.6	Q1
13	Scientific Reports	16	3.8	Q1
14	Journal of Neurology	14	4.8	Q1
15	npj Parkinson's Disease	13	6.7	Q1

Abbreviations: IF, impact factor; JCR, Journal Citation Reports.

**Table 6:** Top 15 cited journals according to co-citations in the co-cited journal network map.

Index	Journal	Publication	Co-citation	IF (2024)
1	Neuroimage	3051	175,181	4.7
2	Movement disorders	2830	167,704	7.4
3	Brain	2147	130,642	10.6
4	Neurology	1440	93,988	7.7
5	Human Brain Mapping	1352	81,525	3.5
6	Parkinsonism and Related Disorders	1116	69,654	3.1
7	PLoS One	810	51,294	2.9
8	Journal of Neurology, Neurosurgery and Psychiatry	779	49,223	8.7
9	Journal of Neuroscience	764	49,014	4.4
10	Proceedings of the National Academy of Sciences	672	41,991	9.4
11	Cerebral Cortex	612	38,840	2.9
12	Journal of Neurology	474	30,445	4.8
13	Annals of Neurology	443	30,651	8.1
14	Neuroscience Letters	443	25,492	2.5
15	Neurobiology of Aging	416	26,113	3.4

Abbreviations: IF, impact factor; JCR, Journal Citation Reports.

## Data Availability

The data that support the findings of this study are available from the corresponding authors upon reasonable request.
